# Synthesis and biological evaluation of some novel coumarin‐oxadiazole conjugates as potential candidates for the treatment of cognitive decline

**DOI:** 10.1002/alz.093546

**Published:** 2025-01-09

**Authors:** Geetakshi Arora, Poonam Piplani

**Affiliations:** ^1^ Panjab University, Chandigarh India; ^2^ Panjab University, chandigarh India

## Abstract

**Background:**

The present study demonstrates the design, synthesis and pharmacological evaluation of coumarin‐oxadiazole hybrids as potential molecules of therapeutic significance for the treatment of cognitive dysfunction.

**Method:**

Eight novel coumarin‐oxadiazole hybrids have been synthesized by employing suitable synthetic procedures and characterized by various spectral techniques i.e. FT‐IR, ^1^H NMR and ^13^C NMR. All synthesized compounds were evaluated for their *in‐vitro* acetylcholinesterase (AChE) inhibition at five different concentrations. After i*n vitro* studies, the compounds have been evaluated against behavioural alterations using step down passive avoidance and escape learning protocol at a dose of 2.0 mg/kg with reference to standard drug, rivastigmine. These compounds were further evaluated for their *ex vivo* acetylcholinesterase inhibition using mice brain homogenate as a source of the enzyme. Biochemical estimation of markers of oxidative stress (lipid peroxidation, superoxide dismutase, glutathione, catalase) has been carried out to access the role of synthesized molecules on the oxidative damage induced by scopolamine.

**Result:**

The compounds 114, 117 and 120 were found to be the most promising in amelioration of cognitive impairment due to scopolamine as evidenced in both the behavioural animal model and *ex vivo* acetylcholinesterase assay. The coumarin‐oxadiazole based analogues have been found to exhibit promising antioxidant effects as indicated by the assessment of various biochemical parameters.

**Conclusion:**

Our results depicted that the anti‐amnesic effect of the synthesized conjugates on scopolamine‐induced memory impairment may be related to the mediation of the cholinergic nervous system and their effective antioxidant activity.

